# Deregulatory miRNA-BDNF Network Inferred from Dynamic Expression Changes in Schizophrenia

**DOI:** 10.3390/brainsci12020167

**Published:** 2022-01-27

**Authors:** Xiaoqian Fu, Yansong Liu, Ancha Baranova, Fuquan Zhang

**Affiliations:** 1 Department of Clinical Psychology, Suzhou Guangji Hospital, The Affiliated Guangji Hospital of Soochow University, Suzhou 215137, China; a126fuxiaoqian@126.com (X.F.); lysway@126.com (Y.L.); 2 School of Systems Biology, George Mason University, Manassas, VA 20110, USA; abaranov@gmu.edu; 3 Research Centre for Medical Genetics, 115478 Moscow, Russia; 4 Institute of Neuropsychiatry, The Affiliated Brain Hospital of Nanjing Medical University, Nanjing 210029, China; 5 Department of Psychiatry, The Affiliated Brain Hospital of Nanjing Medical University, Nanjing 210029, China

**Keywords:** schizophrenia, brain-derived neurotrophic factor, miR-124-3p, miR-132-3p, miR-206, co-expression analyses

## Abstract

(1) Background: Brain-derived neurotrophic factor (BDNF) is one of the promising risk genes for schizophrenia (SZ), a disease with prominent dysregulation of miRNA networks. Here, we present a study of miRNA-BDNF co-expression changes in peripheral blood of SZ patients. (2) Methods: The expression levels of the BDNF mRNA and three validated binding miRNAs—miR-124-3p, miR-132-3p, and miR-206—were quantified in the blood of 48 healthy controls and 32 SZ patients before and after 12 weeks of treatment. The co-expression patterns were evaluated in the three groups. (3) Results: The expression levels of BDNF were significantly downregulated in SZ patients compared to the controls. After the treatment, the expression levels of BDNF were upregulated, while the expression levels of the three miRNAs were downregulated. Co-expression analyses showed positive correlations of this network in the SZ patients, while weak negative correlations were observed in the healthy controls. After the 12-week treatment, the overall correlation between BDNF and the three miRNAs reached the levels comparable to the healthy controls. (4) Conclusions: Our findings suggest the involvement of the miRNA-BDNF network in the onset and treatment of SZ.

## 1. Introduction

Schizophrenia (SZ) is a severe mental disorder characterized by a variety of symptoms, both positive, such as hallucinations and delusion, and negative, such as insufficiency of thinking processes, dull emotional response, reduced will, and cognitive impairment. SZ has an estimated heritability of 60–80% [1,2,3] and leads to significant functional disabilities in one percent of the total world population [4,5]. The disease is believed to have a neurodevelopmental origin, with genetic and environmental factors being etiologically intertwined [6,7].

Brain-derived neurotrophic factor (BDNF) has been widely studied as a biomarker for a range of neuropsychiatric disorders, including SZ [8,9]. BDNF affects cell level maturation, survival, diffusion, and synaptic function by activating intracellular signaling cascades, including mitogen-activated protein kinase/extracellular signal-regulated protein kinase, phosphatidylinositol 3-kinase, and phospholipase C pathways [10,11,12]. BDNF promotes neurogenesis and modulates cognitive function through its effect on synaptic plasticity [13]. Abnormal BDNF expression or function has been repeatedly observed in neurodegenerative and mental diseases.

As a diverse but specific set of mRNA expression silencers, miRNAs regulate the expression of numerous genes and are considered to be vital regulators in neurodevelopment [14,15]. A large number of studies have shown that miRNA expression is abnormal in the brain and the peripheral blood of patients with SZ [16,17]. Several miRNAs, such as miR-124, miR-132, miR-134, and miR-137, have been reported to be associated with SZ [18]. The expression of the BDNF-encoding gene is regulated by a cluster of miRNAs, some of which have been experimentally validated, including miR-1/206 [19], miR-124 [20], let-7d [20], and miR-132-3p [21]. Moreover, miR-132, miR-206, and miR-124 are involved in axonal growth, proliferation, synaptic differentiation, and neurological diseases. As there are few studies on the relationships between BDNF and these miRNAs in SZ, we aimed at discerning the interaction pattern between the levels of BDNF and these miRNAs in this disease.

Previous gene expression studies of SZ typically tested the expression levels of either particular genes or particular miRNAs, while co-expression changes between genes and their binding miRNAs have rarely been examined. Here, we investigate the co-expression patterns between the BDNF-encoding mRNA and its regulatory miRNAs, miR-124-3p, miR-132-3p, and miR-206 in 48 healthy controls as well as 32 SZ patients before and after 12-week of the antipsychotic treatment.

## 2. Materials and Methods

### 2.1. Subjects

We enrolled 48 healthy controls (17 males and 31 females, aged 31.56 ± 6.88 years) and 32 SZ patients (14 males and 18 females, aged 35.84 ± 12.05 years) from the Han Chinese population who were antipsychotic drug-free for at least one month before the enrollment. The average duration of the disease in SZ patients was 130.57 ± 106.71 months. There were no significant differences in gender or age between SZ cases and the healthy controls (Table 1).

The diagnosis of SZ was confirmed in interviews by two or more experienced psychiatrists using the Structured Clinical Interview for DSM-IV (SCID-I) and in line with criteria in the Diagnostic and Statistical Manual of Mental Disorders, Fourth Edition (DSM-IV). Exclusion criteria included the presence of other mood or neurodevelopmental disorders, epilepsy, or intellectual disability. After completing the baseline assessment, SZ patients were treated with one of the following oral atypical antipsychotics, olanzapine (*n* = 10), quetiapine (*n* = 6), aripiprazole (*n* = 6), risperidone (*n* = 5), amisulpride (*n* = 3), or ziprasidone (*n* = 2), and the patients were followed-up after a 12-week period of antipsychotic treatment. The clinical symptoms were assessed by trained psychiatrists with the Positive and Negative Syndrome Scale (PANSS) before and after 12-week treatment. According to the PANSS reductive ratio, 71.8% of patients were responders, and the remaining 28.2% were non-responders. After the antipsychotic treatment, the total scores of PANSS and its subscales (positive scale, negative scale, general psychopathology scale, and supplementary items) were significantly lower than those before treatment (*p* < 0.05) (Table 1). The healthy controls were recruited from local communities or were undergoing routine health check-ups. Subjects with relevant physical diseases or a history of major psychiatric disorders or suicidal behavior were excluded, as well as those who had a first-degree relative with a history of severe mental disorder or suicidal behavior.

The study was approved by the Medical Research Ethics Committee of Wuxi Mental Health Center of Nanjing Medical University, and the code number is 2019-162. Informed consent was signed by either patients or their guardians.

### 2.2. Analysis of Gene Expression by RT-qPCR

A 3 mL sample of peripheral blood was collected from each of the 48 healthy controls and 32 SZ patients before and after antipsychotic treatment. Leukocytes were isolated from the blood by centrifugation. Total RNA was isolated from peripheral blood mononuclear cells (PBMCs) using TRIzol (Invitrogen, Waltham, MA, USA) with on-column DNase I treatment as described by the manufacturer. cDNA was synthesized using a High-Capacity RNA-to-cDNA Kit (Invitrogen, Waltham, MA, USA) as described by the manufacturer. RT–qPCR was performed using the primers listed in Supplementary Table S1. PCR was performed using a 7900HT real-time PCR machine (Applied Biosystems, Waltham, MA, USA) for 2 min at 50 °C, 2 min at 95 °C, and then 40 cycles consisting of 15 s at 95 °C and 60 s at 60 °C, followed by a subsequent standard dissociation protocol to ensure that each amplicon was a single product. All quantifications were made after normalized to GAPDH.

### 2.3. Analysis of miRNA Expression by RT-qPCR

Total RNA was isolated from PBMCs using TRIzol (Invitrogen, Waltham, MA, USA) with on-column DNase I treatment as described by the manufacturer. Analysis of miRNAs was performed using the miScript system (QIAGEN, Toronto, ON, Canada) (including miScript Reverse Transcription kit, miScript Primer Assays, and miScript SYBR Green PCR kit) as described by the protocol provided by the company. RT-qPCR was performed using the primer listed in Supplementary Table S1. Small nuclear RNA U6 was used for normalization. RT-qPCR was conducted using a standard SYBR Green protocol on an Applied Biosystems 7900HT Sequence Detection System (Applied Biosystems). The reactions were incubated at 95 °C for 15 min, followed by 40 cycles of 94 °C for 15 s, 55 °C for 30 s, and 70 °C for 34 s. All reactions were run in triplicate. The threshold cycle (CT) is defined as the fractional cycle number at which the fluorescence passes the fixed threshold.

### 2.4. Statistical and Bioinformatics Analysis

The differences in gene expression levels between the patient and control groups were analyzed by the Mann–Whitney U test using R because the expression levels were not normally distributed. Paired Mann–Whitney U tests were used to compare expression levels in samples collected from SZ patients before and after antipsychotic treatment. The partial Spearman coefficient of correlation adjusted by sex and age was calculated using the R package ppcor [22], and the differential test of coefficient of correlation was analyzed using Fisher’s Z-transformation implemented in the R package DiffCorr [23].

## 3. Results

### 3.1. Comparing Expression Levels in SZ Patients and Healthy Controls

**Figure 1:**
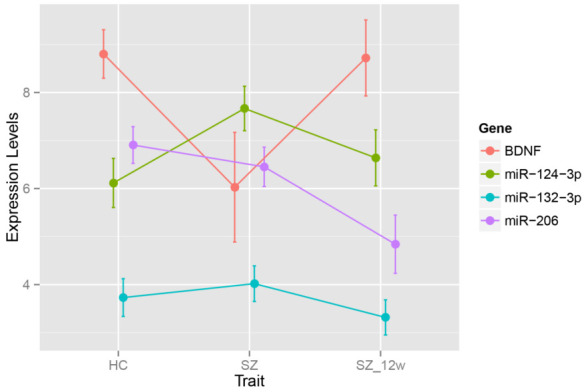
Expression levels in healthy controls and SZ patients before and after antipsychotic treatment. HC—healthy control; SZ—schizophrenia.

In SZ cases, the BDNF expression levels were downregulated, while miR-124-3p levels were increased (FDR < 0.05) compared to those in the controls. There were no significant differences in miR-206 and miR-132-3p levels between SZ cases and the controls (Table 2 and Figure 1).

### 3.2. Comparing Expression Levels in SZ Patients before and after Antipsychotic Treatment

After the 12-week antipsychotic treatment, the levels of BDNF were upregulated, while the levels of three miRNAs (miR-124-3p, miR-206, and miR-132-3p) were all downregulated (FDR < 0.05) (Table 3, Figure 1).

### 3.3. Co-Expression Analysis in SZ Patients and Healthy Controls

**Figure 2:**
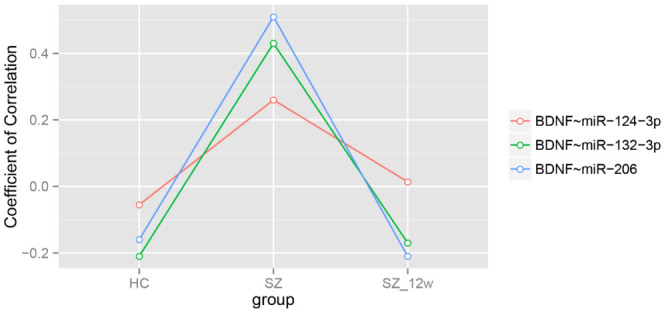
Partial Spearman correlation coefficients adjusted by age and sex.

After adjustment for age and sex, the correlations among the levels of BDNF mRNA and the miRNAs were negative in the healthy controls (FDR < 0.05). The correlation coefficients between BDNF and each of the three miRNAs were all positive, with BDNF~miR-132-3p and BDNF~miR-206 being statistically significant (FDR < 0.05) (Table 4 and Figure 2).

### 3.4. Co-Expression Analysis in SZ Patients before and after Antipsychotic Treatment

After the 12-week antipsychotic treatment, the overall correlation coefficient between BDNF and the three miRNAs were decreased, comparable to those in the healthy controls, predominantly due to the correlation changes in the BDNF~miR-132-3p and BDNF~miR-206 pairs (FDR < 0.05) (Table 4 and Figure 2).

## 4. Discussion

In our study, the expression levels of BDNF were significantly downregulated in SZ patients when compared to that in controls. Notably, the antipsychotic treatment led to an increase in the levels of the BDNF-encoding mRNA. In patients with the first episode of SZ and those on chronic medication, Favalli et al. [24] found a significant reduction in serum BDNF expression in comparison to that observed in healthy controls. Another study also showed reduced levels of BDNF expression in serum samples of patients with chronic SZ [25].

Our study also confirms previous findings that the expression of BDNF may be induced by antipsychotics. A Spanish study found that during the first psychotic episode, the levels of BDNF in plasma are lower than those in healthy controls and that the antipsychotic treatment restores the levels of BDNF to the levels seen in healthy controls [26]. Moreover, one meta-analysis further showed that the serum levels of BDNF in patients with SZ were reduced independently of whether they were treated or not [27]. The study conducted in Japan showed that eight weeks of antipsychotic treatment failed to alter the levels of BDNF in plasma of the first-episode SZ patients [28]. Later observations are inconsistent with our own, possibly due to the difference in the duration of the treatment, ethnicity, drug type, drug dose, sample size, or the source of samples (plasma or serum).

Many lines of evidence indicate that the abnormal expression of miRNA is associated with neurodegeneration and the development of neuropsychiatric diseases [29,30,31,32]. In particular, miRNA-mediated dysregulation of the genetic networks is increasingly considered to be related to the etiology of SZ [33,34]. miR-132 is involved in the processes of axonal growth, proliferation, and synaptic differentiation, in part, through the regulation of the function of BDNF [35,36]. miR-124 is the most abundant miRNA in the brain [37], where it plays an important role in neurite outgrowth through regulating the expression of several protein signals, including BDNF [38,39,40]. In our study of SZ patients, miR-124-3p levels were upregulated, while BDNF expression was downregulated when compared to those in the controls. After the treatment, the miR-124-3p expression was downregulated, while the BDNF expression was upregulated. Our study showed that after antipsychotic treatment, the expression levels of BDNF and miR-124-3p returned to a negatively correlated pattern, which is considered to be the normal state.

Co-expression analysis of our study shows weak negative correlations between the levels of mRNA for *BDNF* and the respective miRNAs in healthy controls, consistent with the typical post-transcriptionally repressive effect of miRNAs on their target genes. Notably, in SZ patients, the correlations of *BDNF* mRNA levels with those of the miRNAs were positive, indicating that the negative regulatory relationships between miRNAs and BDNF diminished. This loss of regulatory effects of the miRNAs on BDNF may contribute to the development of SZ. The reported observations may also be possibly explained by the changes in levels or activity of other, non-miRNA-based regulations of the BDNF expression. For example, long non-coding RNA (lncRNAs) also modulate levels of individual protein-coding transcripts, acting through a variety of mechanisms, including the sponging of miRNAs [41], generation of new miRNAs [42], induction of genomic imprinting [43], and so on. Badrlou et al. [44] found that the levels of BDNF mRNA and three BDNF-associated lncRNAs (BDNF-AS, MIR137HG, and MIAT) in peripheral blood could discriminate SZ patients from normal subjects with a diagnostic power of 71%, 72%, 67%, and 68%, respectively. Increasing evidence supports the potential involvement of lncRNAs in SZ [45,46,47].

After 12 weeks of antipsychotic treatment, the co-expression patterns between BDNF and its binding miRNAs were restored to the normal state of being weakly negative. The treatment incurred alterations in both the levels of BDNF-encoding mRNA and, more importantly, in overall correlations within the miRNA-BDNF network. In this regard, it is interesting that Sun et al. [48] found that the expression levels of miR-30e, miR-181b, miR-34a, miR-346, and miR-7, when measured as a group, were significantly higher in SZ patients as compared to healthy controls, suggesting that the overall changes in the levels of these miRNAs produced diagnostic value when they were looked at as a set of biomarkers, rather than individual ones. After drug treatment, the expression levels of these miRNAs were significantly reduced, and the improvement of clinical symptoms was significantly correlated with observed changes. This observation was consistent with our results and further supports the notion that the changes in miRNAs levels may be directly influenced by drug intervention.

A thorough understanding of transcription factors-miRNA-target gene axis may provide important clues to the molecular pathogenesis of SZ [16,49]. Of note, alterations within co-expression networks may occur with or without respective changes in the levels of its constituents. Our findings suggest that the dynamic pattern of RNAs encoding BDNF and its regulators may serve as a more reliable indicator of SZ-related pathological changes than BDNF itself. Antipsychotic therapy may exert its effects by regulating the balance of individual molecules within the miRNA-BDNF network.

The consistency of the changes observed in the co-expression relationships of BDNF with miR-132-3p and miR-206 further supports the hypothesis describing SZ phenotype in terms of dysregulation in a group of miRNAs, rather than a result of an alteration in the level of a particular miRNAs. As one miRNA may bind many targets, BDNF is not the only molecule affected by each miRNA.

Several limitations need to be pointed out. First, due to the rarity of the samples from antipsychotic drug-free SZ patients, the sample size in our study was relatively small. Second, as the findings of our study were derived from the PBMCs, care should be taken when comparing these data with observations in brains. In addition, the SZ patients were using different types of antipsychotics which could have influenced the results, even if all these antipsychotics were of the atypical group. This factor should be minimized as much as possible in the future. Some biological markers may change seasonally or due to environmental factors in healthy participants [50], including the BDNF protein [51]. Caution should be taken that only the SZ patients were followed up after the 12-week treatment period in the study. Due to the lack of follow-up in the controls, we could not exclude the seasonal or environmental factors that could affect BDNF in controls over the 12-week period, which certainly warrants further investigation with longitudinal control cohorts. Indeed, serum levels of BDNF were unequivocally shown to vary over the year systematically, depending on exposure to sunlight [51]. Moreover, one of the miRNAs profiled in the current study, namely, miR-132 is involved in photoperiodic regulation, at least in rodents [52]. Finally, the demographic data we collected were not comprehensive enough. For example, we could not assess whether smoking affects the expression patterns of the mRNA or miRNAs.

## 5. Conclusions

Our study supports the involvement of deregulated miRNA-BDNF network in the pathophysiology of SZ.

## Figures and Tables

**Table 1:** Demographics of SZ patients and healthy controls.

Variable	HC (*n* = 48)	SZ (*n* = 32)	SZ_12w (*n* = 32)	t/χ^2^	*p*
Gender (M/F)	17/31	14/18		0.56	0.45
Age	31.56 ± 6.88	35.84 ± 12.05		−1.89	0.07
Ethnicity	Han	Han			
Years of education		8.65 ± 2.85			
Age at onset		26.28 ± 10.47			
Duration of illness (month)		130.57 ± 106.71			
First onset		7			
Recent onset (≤60 mouths)		7			
Chronic (>60 mouths)		18			
PANSS score (total)		102.59 ± 15.53	63.07 ± 14.37	12.03	<0.01
PANSS score (P)		23.36 ± 9.14	10.88 ± 3.55	6.77	<0.01
PANSS score (N)		24.40 ± 8.22	18.44 ± 5.77	4.43	<0.01
PANSS score (G)		46.20 ± 7.44	31.24 ± 6.48	8.82	<0.01
PANSS score (S)		8.24 ± 2.73	3.88 ± 1.74	6.41	<0.01

HC—healthy control; SZ—schizophrenia; SZ_12w—schizophrenia after 12-week treatment; M—male; F—female; Han—Han Chinese population; PANSS—Positive and Negative Syndrome Scale; P—positive scale; N—negative scale; G—general psychopathology scale; S—supplementary items.

**Table 2:** Expression levels in healthy controls and in SZ patients.

Gene	HC (*n* = 48)	SZ (*n* = 32)	FC	*p*	FDR
*BDNF*	8.80 ± 1.74	6.03 ± 3.00	0.24	5.72 × 10^−6^	1.14 × 10^−5^
*miR-124-3p*	6.12 ± 1.76	7.67 ± 1.28	2.18	4.73 × 10^−6^	1.14 × 10^−5^
*miR-206*	6.91 ± 1.31	6.45 ± 1.14	0.61	0.08	0.11
*miR-132-3p*	3.73 ± 1.35	4.02 ± 1.03	1.12	0.70	0.70

HC—healthy control; SZ—schizophrenia; FC—fold change; FDR—False Discovery Rate.

**Table 3:** Expression levels in SZ patients before and after antipsychotic treatment.

Gene	SZ (*n* = 32)	SZ_12w (*n* = 32)	FC	*p*	FDR
*BDNF*	6.03 ± 3.00	8.72 ± 2.12	3.68	3.78 × 10^−4^	7.55 × 10^−4^
*miR-124-3p*	7.67 ± 1.28	6.64 ± 1.62	0.62	7.28 × 10^−4^	9.71 × 10^−4^
*miR-206*	6.45 ± 1.14	4.84 ± 1.68	0.44	2.50 × 10^−5^	1.00 × 10^−4^
*miR-132-3p*	4.02 ± 1.03	3.32 ± 1.01	0.64	2.54 × 10^−3^	2.54 × 10^−3^

SZ—schizophrenia; SZ_12w—schizophrenia after 12-week treatment; FC—fold change; FDR—False Discovery Rate.

**Table 4:** Co-expression analysis in healthy controls and SZ patients before and after antipsychotic treatment.

Correlation	HC	SZ	SZ_12w	P__SZ/HC_	FDR__SZ/HC_	P__SZ_12w/SZ_	FDR__SZ_12w/SZ_
*BDNF~miR-124-3p*	−0.06	0.26	0.01	0.190	0.190	0.350	0.350
*BDNF~miR-206*	−0.16	0.51	−0.21	3.20 × 10^−3^	0.013	4.56 × 10^−3^	0.018
*BDNF~miR-132-3p*	−0.21	0.43	−0.17	6.39 × 10^−3^	0.013	0.021	0.042
*BDNF~all miRNAs*	−0.11	0.21	−0.07	0.020	0.027	0.069	0.092

HC—healthy control; SZ—schizophrenia; SZ_12w—schizophrenia after 12-week treatment; P__SZ/HC_—*p* value of SZ and HC co-expression analysis; FDR__SZ/HC_—False Discovery Rate of SZ and HC co-expression analysis; P__SZ_12w/SZ_—*p* value of SZ_12w and SZ co-expression analysis; FDR__SZ_12w/SZ_—False Discovery Rate of SZ_12w and SZ co-expression analysis; BDNF—brain-derived neurotrophic factor.

## Data Availability

For access to the data in this paper, interested researchers may contact the corresponding author via email: zhangfq@njmu.edu.cn.

## Supplementary Materials

The following are available online at https://www.mdpi.com/article/10.3390/brainsci12020167/s1, Table S1: Primers materials.

## References

[1] Meyer-Lindenberg A. (2011). Neuroimaging and the question of neurodegeneration in schizophrenia. Prog. Neurobiol..

[2] Sullivan P.F. (2005). The genetics of schizophrenia. PLoS Med..

[3] Battle D.E. (2013). Diagnostic and Statistical Manual of Mental Disorders (DSM). CoDAS.

[4] Perkins D.O., Jeffries C.D., Jarskog L.F., Thomson J.M., Woods K., Newman M.A., Parker J.S., Jin J., Hammond S.M. (2007). microRNA expression in the prefrontal cortex of individuals with schizophrenia and schizoaffective disorder. Genome Biol..

[5] Rössler W., Salize H.J., van Os J., Riecher-Rössler A. (2005). Size of burden of schizophrenia and psychotic disorders. Eur. Neuropsychopharmacol..

[6] Lichtenstein P., Björk C., Hultman C.M., Scolnick E., Sklar P., Sullivan P.F. (2006). Recurrence risks for schizophrenia in a Swedish National Cohort. Psychol. Med..

[7] Sullivan P.F., Kendler K.S., Neale M.C. (2003). Schizophrenia as a Complex Trait: Evidence from a meta-analysis of twin studies. Arch. Gen. Psychiatry.

[8] Harrisberger F., Smieskova R., Schmidt A., Lenz C., Walter A., Wittfeld K., Grabe H.J., Lang U.E., Fusar-Poli P., Borgwardt S. (2015). BDNF Val66Met polymorphism and hippocampal volume in neuropsychiatric disorders: A systematic review and meta-analysis. Neurosci. Biobehav. Rev..

[9] Fu X., Wang J., Du J., Sun J., Baranova A., Zhang F. (2020). BDNF Gene’s Role in Schizophrenia: From Risk Allele to Methylation Implications. Front. Psychiatry.

[10] Minichiello L. (2009). TrkB signalling pathways in LTP and learning. Nat. Rev. Neurosci..

[11] Russo S.J., Mazei-Robison M.S., Ables J.L., Nestler E.J. (2009). Neurotrophic factors and structural plasticity in addiction. Neuropharmacology.

[12] Numakawa T., Suzuki S., Kumamaru E., Adachi N., Richards M., Kunugi H. (2010). BDNF function and intracellular signaling in neurons. Histol. Histopathol..

[13] Lee S.-T., Chu K., Jung K.-H., Kim J.H., Huh J.-Y., Yoon H., Park D.-K., Lim J.-Y., Kim J.-M., Jeon D. (2012). miR-206 regulates brain-derived neurotrophic factor in Alzheimer disease model. Ann. Neurol..

[14] Li X., Jin P. (2010). Roles of small regulatory RNAs in determining neuronal identity. Nat. Rev. Neurosci..

[15] Im H.-I., Kenny P.J. (2012). MicroRNAs in neuronal function and dysfunction. Trends Neurosci..

[16] Ghafouri-Fard S., Eghtedarian R., Taheri M., Brühl A.B., Sadeghi-Bahmani D., Brand S. (2021). A Review on the Expression Pattern of Non-Coding RNAs in Patients with Schizophrenia: With a Special Focus on Peripheral Blood as a Source of Expression Analysis. Front. Psychiatry.

[17] Xu Y., Yue W., Shugart Y.Y., Li S., Cai L., Li Q., Cheng Z., Wang G., Zhou Z., Jin C. (2015). Exploring Transcription Factors-microRNAs Co-regulation Networks in Schizophrenia. Schizophr. Bull..

[18] Mellios N., Sur M. (2012). The Emerging Role of microRNAs in Schizophrenia and Autism Spectrum Disorders. Front. Psychiatry.

[19] Lewis B.P., Shih I.-h., Jones-Rhoades M.W., Bartel D.P., Burge C.B. (2003). Prediction of mammalian microRNA targets. Cell.

[20] Chandrasekar V., Dreyer J.-L. (2009). microRNAs miR-124, let-7d and miR-181a regulate Cocaine-induced Plasticity. Mol. Cell. Neurosci..

[21] Klein M.E., Lioy D.T., Ma L., Impey S., Mandel G., Goodman R.H. (2007). Homeostatic regulation of MeCP2 expression by a CREB-induced microRNA. Nat. Neurosci..

[22] Kim S. (2012). Ppcor: Partial and Semi-Partial (Part) Correlation. http://CRAN.R-project.org/package=ppcor.

[23] Fukushima A., Nishida K. (2015). DiffCorr: Analyzing and Visualizing Differential Correlation Networks in Biological Data. http://CRAN.R-project.org/package=DiffCorr.

[24] Favalli G., Li J., Belmonte-De-Abreu P., Wong A.H.C., Daskalakis Z.J. (2012). The role of BDNF in the pathophysiology and treatment of schizophrenia. J. Psychiatr. Res..

[25] Xiu M.H., Hui L., Dang Y.F., De Hou T., Zhang C.X., Zheng Y.L., Chen D.C., Kosten T.R., Zhang X.Y. (2009). Decreased serum BDNF levels in chronic institutionalized schizophrenia on long-term treatment with typical and atypical antipsychotics. Prog. Neuro-Psychopharmacol. Biol. Psychiatry.

[26] González-Pinto A., Mosquera F., Palomino A., Alberich S., Gutiérrez A., Haidar K., Vega P., Barbeito S., Ortiz A., Matute C. (2010). Increase in brain-derived neurotrophic factor in first episode psychotic patients after treatment with atypical antipsychotics. Int. Clin. Psychopharm..

[27] Green M.J., Matheson S.L., Shepherd A., Weickert C.S., Carr V.J. (2010). Brain-derived neurotrophic factor levels in schizophrenia: A systematic review with meta-analysis. Mol. Psychiatry.

[28] Yoshimura R., Ueda N., Hori H., Ikenouchi-Sugita A., Umene-Nakano W., Nakamura J. (2010). Different patterns of longitudinal changes in plasma levels of catecholamine metabolites and brain-derived neurotrophic factor after administration of atypical antipsychotics in first episode untreated schizophrenic patients. World J. Biol. Psychiatry.

[29] Miller B.H., Wahlestedt C. (2010). MicroRNA dysregulation in psychiatric disease. Brain Res..

[30] Xu B., Karayiorgou M., Gogos J.A. (2010). MicroRNAs in psychiatric and neurodevelopmental disorders. Brain Res..

[31] Welberg L. (2010). Neurodegenerative disorders: Reconnect with microRNA. Nat. Rev. Neurosci..

[32] Hebert S.S., De Strooper B. (2009). Alterations of the microRNA network cause neurodegenerative disease. Trends Neurosci..

[33] Zhang F., Xu Y., Shugart Y.Y., Yue W., Qi G., Yuan G., Cheng Z., Yao J., Wang J., Wang G. (2014). Converging Evidence Implicates the Abnormal MicroRNA System in Schizophrenia. Schizophr. Bull..

[34] Cao H., Baranova A., Yue W., Yu H., Zhu Z., Zhang F., Liu D. (2020). miRNA-Coordinated Schizophrenia Risk Network Cross-Talk with Cardiovascular Repair and Opposed Gliomagenesis. Front. Genet..

[35] Numakawa T., Richards M., Adachi N., Kishi S., Kunugi H., Hashido K. (2011). MicroRNA function and neurotrophin BDNF. Neurochem. Int..

[36] Wanet A., Tacheny A., Arnould T., Renard P. (2012). miR-212/132 expression and functions: Within and beyond the neuronal compartment. Nucleic Acids Res..

[37] Sonntag K.C., Woo T.-U.W., Krichevsky A.M. (2012). Converging miRNA functions in diverse brain disorders: A case for miR-124 and miR-126. Exp. Neurol..

[38] Vo N., Klein M.E., Varlamova O., Keller D.M., Yamamoto T., Goodman R.H., Impey S. (2005). From The Cover: A cAMP-response element binding protein-induced microRNA regulates neuronal morphogenesis. Proc. Natl. Acad. Sci. USA.

[39] Chandrasekar V., Dreyer J.-L. (2011). Regulation of MiR-124, Let-7d, and MiR-181a in the Accumbens Affects the Expression, Extinction, and Reinstatement of Cocaine-Induced Conditioned Place Preference. Neuropsychopharmacology.

[40] Bahi A., Dreyer J.-L. (2013). Striatal modulation of BDNF expression using microRNA124a-expressing lentiviral vectors impairs ethanol-induced conditioned-place preference and voluntary alcohol consumption. Eur. J. Neurosci..

[41] Paraskevopoulou M.D., Hatzigeorgiou A.G. (2016). Analyzing MiRNA–LncRNA Interactions. Methods Mol. Biol..

[42] Rodriguez A., Griffiths-Jones S., Ashurst J.L., Bradley A. (2004). Identification of Mammalian microRNA Host Genes and Transcription Units. Genome Res..

[43] Magistri M., Faghihi M.A., Laurent G.S., Wahlestedt C. (2012). Regulation of chromatin structure by long noncoding RNAs: Focus on natural antisense transcripts. Trends Genet..

[44] Badrlou E., Ghafouri-Fard S., Omrani M.D., Neishabouri S.M., Arsang-Jang S., Taheri M., Pouresmaeili F. (2021). Expression of BDNF-Associated lncRNAs in Treatment-Resistant Schizophrenia Patients. J. Mol. Neurosci..

[45] Liu S., Rao S., Xu Y., Li J., Huang H., Zhang X., Fu H., Wang Q., Cao H., Baranova A. (2019). Identifying common genome-wide risk genes for major psychiatric traits. Hum. Genet..

[46] Wu Y., Cao H., Baranova A., Huang H., Li S., Cai L., Rao S., Dai M., Xie M., Dou Y. (2020). Multi-trait analysis for genome-wide association study of five psychiatric disorders. Transl. Psychiatry.

[47] Liu Y., Rao S., Xu Y., Zhang F., Wang Z., Zhao X. (2018). Changes in the level of Long Non-Coding RNA Gomafu gene expression in schizophrenia patients before and after antipsychotic medication. Schizophr. Res..

[48] Sun X.-Y., Zhang J., Niu W., Guo W., Song H.-T., Li H.-Y., Fan H.-M., Zhao L., Zhong A.-F., Dai Y.-H. (2015). A preliminary analysis of microRNA as potential clinical biomarker for schizophrenia. Am. J. Med. Genet. Part B Neuropsychiatr. Genet..

[49] Liu S., Zhang F., Shugart Y.Y., Yang L., Li X., Liu Z., Sun N., Yang C., Guo X., Shi J. (2017). The early growth response protein 1-miR-30a-5p-neurogenic differentiation factor 1 axis as a novel biomarker for schizophrenia diagnosis and treatment monitoring. Transl. Psychiatry.

[50] Smith M.N., Wilder C.S., Griffith W.C., Workman T., Thompson B., Dills R., Onstad G., Vredevoogd M., Vigoren E.M., Faustman E.M. (2015). Seasonal variation in cortisol biomarkers in Hispanic mothers living in an agricultural region. Biomarkers.

[51] Molendijk M.L., Haffmans J.P.M., Bus B.A.A., Spinhoven P., Penninx B.W.J.H., Prickaerts J., Voshaar R.C.O., Elzinga B.M. (2012). Serum BDNF Concentrations show strong seasonal variation and correlations with the amount of ambient sunlight. PLoS ONE.

[52] Mendoza-Viveros L., Chiang C.-K., Ong J.L., Hegazi S., Cheng A.H., Bouchard-Cannon P., Fana M., Lowden C., Zhang P., Bothorel B. (2017). miR-132/212 Modulates seasonal adaptation and dendritic morphology of the central circadian clock. Cell Rep..

